# Parathyroid involvement in thyroid cancer: an unforeseen event

**DOI:** 10.1186/1477-7819-10-121

**Published:** 2012-06-28

**Authors:** Alexandra Chrisoulidou, Stylianos Mandanas, Periklis Mitsakis, Paschalia K Iliadou, Kosmas Manafis, Nikolaos Flaris, Maria Boudina, Lemonia Mathiopoulou, Kalliopi Pazaitou-Panayiotou

**Affiliations:** 1 Department of Endocrinology & Endocrine Oncology, Theagenio Cancer Hospital, 2 Al Simeonidi street, Thessaloniki, 54007, Greece; 2 Pathology Department, General Hospital of Kavala, 65302, Kavala, Greece; 3 Pathology Department, Hippokratio Hospital, Thessaloniki, Greece

**Keywords:** Metastasis, Parathyroid, Thyroid cancer

## Abstract

**Background:**

Parathyroid metastatic disease from thyroid cancer has not been studied extensively, mainly due to the need for parathyroid preservation during thyroid surgery.

**Methods:**

We reviewed files from 1,770 patients with thyroid cancer followed up in our department and 10 patients with parathyroid metastases (0.5%) were identified. Patient and tumor characteristics were recorded.

**Results:**

Six out of ten patients had metastases from papillary thyroid cancer, three from follicular thyroid cancer and one from anaplastic thyroid cancer. In nine patients parathyroid infiltration from thyroid cancer was found in direct contact with the thyroid cancer, and in one patient metastatic foci were observed not in continuity with the thyroid cancer.

**Conclusions:**

Parathyroid involvement, although infrequent, may occur in thyroid cancer independently of patient age and tumor size. The clinical significance of such event is not clear. The influence on disease outcome remains to be elucidated.

## Background

The parathyroid glands are rare metastatic sites from different cancers such as bronchogenic carcinomas, leukemia, thyroid and breast cancers [1] and the earlier reports regarding parathyroid involvement included cases from postmortem series [2]. Largely, the incidence of parathyroid metastatic disease in thyroid cancer is unknown, since infiltration of the parathyroid glands is not sought during surgery, even in invasive tumors, and published data on parathyroid involvement in thyroid cancer are scant. A few reports on parathyroid invasion or metastases from thyroid cancer exist only for papillary thyroid cancer (PTC) [3].

Parathyroid metastatic disease in thyroid cancer may occur via two different pathways. Most commonly the tumor infiltrates the thyroid capsule and invades the parathyroid due to direct contact; less often a hematogenous spread occurs and metastatic foci appear in the parathyroid gland [4].

We aimed to select those cases from our cohort patients with thyroid cancer that had parathyroid infiltration. We recognized parathyroid metastases in PTC, follicular thyroid cancer (FTC) and anaplastic thyroid cancer patients. As reports do not exist for parathyroid involvement in FTC, we described in more detail the characteristics of these cases.

## Methods

All files from thyroid cancer patients (1,770 in total) followed up at the Department of Endocrinology & Endocrine Oncology in Theagenio Cancer Hospital of Thessaloniki, Greece, were retrospectively reviewed. Ten patients with parathyroid involvement were identified from the histological reports. Clinicopathological data including sex, age at diagnosis, size and histological type, tumoral characteristics and extent of metastatic disease of thyroid cancer were recorded for every patient. The study was approved by the Institutional Review Board.

Parathyroid involvement was defined as the infiltration of the thyroid tumor into the parathyroid gland, in the presence or absence of direct invasion from the primary tumor.

Post-surgically, thyroid gland surgical specimens were fixed in formaldehyde 10%, multiple areas of the gland, tumor and capsule were sampled, embedded in paraffin and cut in 4 μ width sections. Staining methods were H&E and immunohistochemistry with thyroid transcription factor-1.

## Results

In our cohort of 1,770 patients with thyroid cancer, 254 had histologically proven parathyroid removal at the time of thyroidectomy (in 225 a single parathyroid gland was removed and in 29 patients two parathyroid glands were removed). As a result, the frequency of parathyroid involvement from thyroid cancer among cases that parathyroid were excised was 3.94% (10 out of 254 cases). In total the frequency of parathyroid involvement in thyroidectomised patients was 0.5% (10 out of 1770 patients).

Six patients were identified with parathyroid involvement from PTC, three from FTC and one from anaplastic thyroid cancer. Clinical and histological characteristics are shown in Table 1. The mode of involvement was invasion to the parathyroid gland from the adjacent thyroid cancer in nine cases and metastasis to the parathyroid gland in one case with FTC. The size of the primary thyroid tumor had a median diameter of 2.5 (range 1 to 5) cm. In the nine cases with parathyroid invasion, extrathyroidal extension to soft tissues was also identified, as shown in Table 1. None of the patients presented with extrathyroidal extension to trachea, oesophagus or jugular vein.

**Table 1:** Clinical and histological characteristics of the patients

**Patient**	**Age**	**Sex**	**Stage**	**Type of TC**	**Cervical infiltration**	**LN meta-**	**Distant meta-**	**Follow-up (years)**
1^*^	62	f	IV	FTC	F, V, M	no	L, B	7
2	57	f	III	FTC	C, V	no	no	10
3	55	f	III	FTC	C	no	no	4.5
4	56	m	III	PTC	C	yes	no	3
5	42	f	I	PTC	C, F	yes	no	2.5
6	71	f	III	PTC	C, F, N, M	yes	no	1
7	17	m	II	PTC	C, F, N, M	yes	L	1
8	18	f	I	PTC	C, F	no	no	11
9	68	f	III	PTC	C, F, N, M	no	no	1
10	76	f	IV	ATC	C, F, N, M	yes	no	1

TC, thyroid cancer; LN, lymph node; f, female, m, male; FTC, follicular thyroid cancer; PTC, papillary thyroid cancer; ATC, anaplastic thyroid cancer; F, fat tissue; V, vessels; M, muscle; C, thyroid capsule; N, nerves; L, lung; B, brain. ^*^Patient 1 is deceased because of brain metastases.

Eight out of ten patients had focal infiltration (≤50% of the parathyroid gland) rather than involvement of the entire gland (which was seen in two patients). In nine patients only one parathyroid was excised and the remaining patient had two glands removed, one infiltrated and one normal parathyroid gland. In all patients one metastatic parathyroid gland was found. In the reported cases, parathyroid glands were removed mostly accidentally during thyroidectomy.

**Figure 1:**
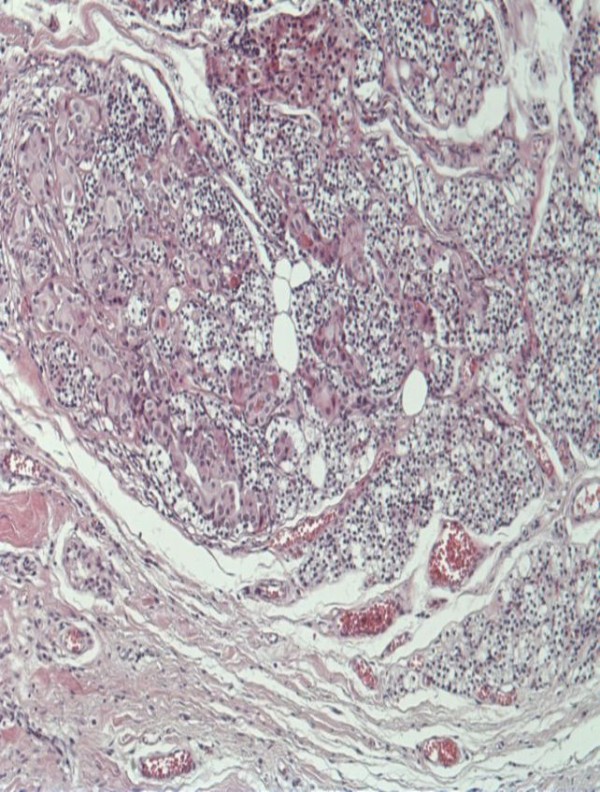
**H&E stained section of affected parathyroid gland (x100).** A parathyroid gland is shown, invaded from a follicular thyroid carcinoma. Malignant cells are large, composed of eosinophilic cytoplasm and round nuclei. These tumor cells form groups or scant follicles with parathyroid cells and lipocytes among them. At the periphery of this picture the parathyroid capsule is shown.

**Figure 2:**
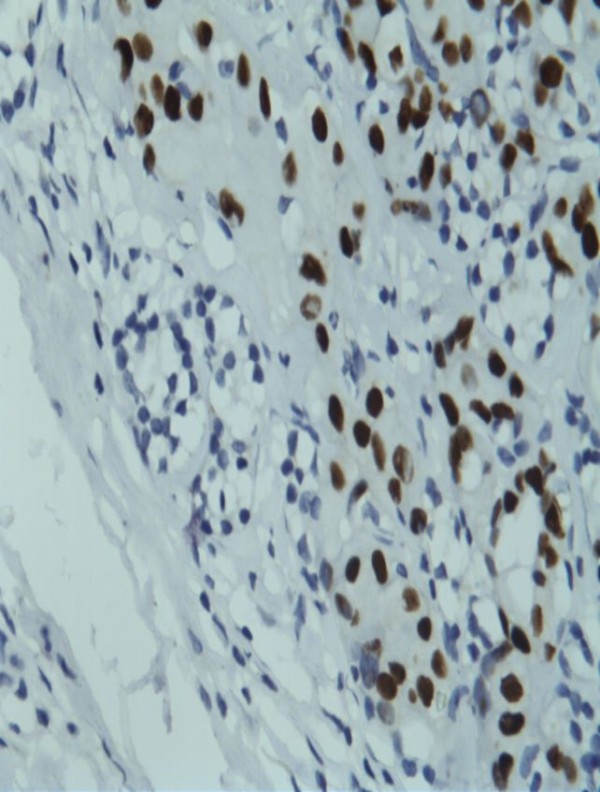
**Positive immunohistochemical staining of the parathyroid gland (shown in** Figure 1**) for thyroid transcription factor-1.** Tumor cells have dark nuclei while parathyroid cells are negative with clear cytoplasm.

**Figure 3:**
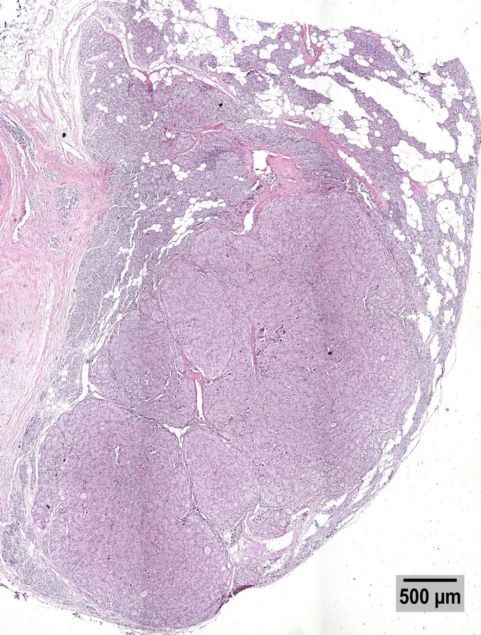
**Low power picture of an H&E stained section of the parathyroid with an intraparenchymal metastasis.** The lobe of the thyroid is infiltrated by a well demarcated, irregularly circumscribed, multinodular, solid, tan neoplasm with microscopic characteristics of poorly differentiated follicular carcinoma, which shows extensive vascular invasion. A grossly identified parathyroid on the surface of the lobe shows infiltration by the carcinoma, not in direct continuity with the tumor of the thyroid. This is a 500 μm calibrated picture.

**Figure 4:**
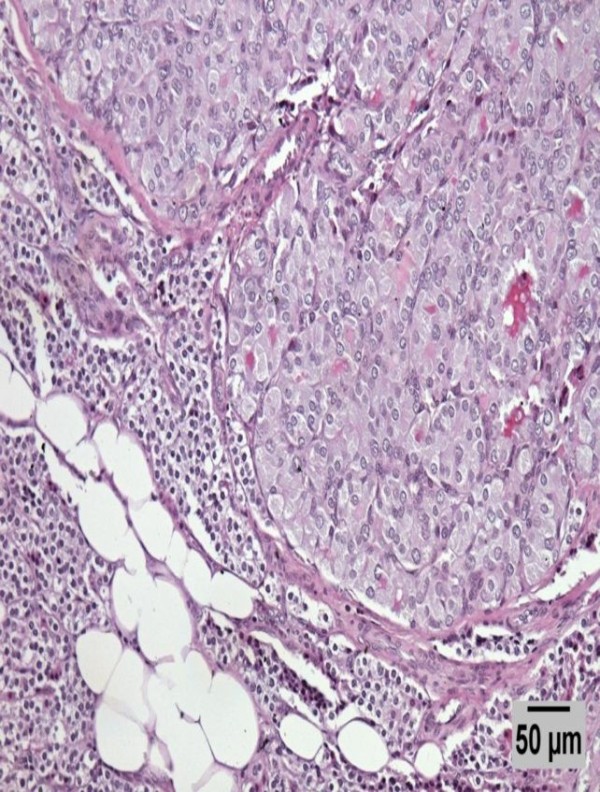
**Medium power picture of an H&E stained section of the parathyroid intraparenchymal metastatic nodule.** A higher magnification picture of the tumor in the affected parathyroid (50 μm calibrated picture).

Due to differences in clinical outcome, and despite the small sample size, the cases of FTC and PTC were reviewed separately. All the PTC patients with parathyroid involvement had invasion of the thyroid capsule, and in 70% of patients lymph node metastases were also found. In the three FTC patients, however, lymph node metastases were not observed, but all demonstrated extrathyroidal extension. Four out of six PTC patients, but none of the FTC patients, had multifocal tumors. The histological features of two out of three cases with FTC are shown in Figures 1, 2, 3 and 4. Lung metastases developed in a 17-year-old male with PTC and one FTC patient (who also developed brain metastases during follow-up). The patient with the anaplastic thyroid cancer was a 75-year-old female with locally invasive tumor and parathyroid, lymph node and vessel infiltration.

Post-surgical median (range) serum calcium levels were 9 (7.3 to 10) mg/dl. Transient hypoparathyroidism after surgery was seen in two patients and permanent hypoparathyroidism was seen in one patient, treated with calcium and vitamin D supplementation.

## Discussions

The parathyroid involvement is an uncommon event in patients with thyroid cancer. In PTC, incidence rates of 1.6 to 2.2% are reported [3;4]. Among patients with histologically proven parathyroid examination after thyroidectomy, the rate of parathyroid involvement is much higher (7.9%) [5]. From our cohort, the frequency of parathyroid involvement is very low (0.5%), and among cases with removed parathyroid glands is 3.95% as only a few patients had parathyroid removal at the time of thyroid surgery. Probably this frequency does not reflect the true parathyroid involvement. In surgical practice, even when extensive surgery is planned for a patient with thyroid cancer, special attention is paid to the preservation of parathyroid glands. As a result, the presence of parathyroid involvement identified during thyroidectomy is probably underestimated and the true extent is unknown. Parathyroid invasion has been associated with advanced stage of disease and lung metastases in older male patients [5]. Invasion of adjacent structures was seen in all our patients, in accordance with previous reports. However, old age was not a significant factor in our cases, as two patients were 17 and 18 years old at the time of diagnosis.

Published data up to now originated only from Japan [3-5]. There are no European or American reports, except from isolated reports [6], to identify the extent of this phenomenon in other populations and to seek possible regional differences. Therefore, the current staging system for differentiated thyroid cancer does not include the parathyroid as a metastatic site [7], possibly due to the rarity of the event.

In all our patients, only one parathyroid gland was found affected in the histological material. Therefore, the presence of micro-metastases in the remaining glands is possible. Microscopic residual disease in advanced thyroid cancer has better prognosis in terms of recurrence and survival compared with macroscopic disease that is left post-surgically [8]. When a preoperative diagnosis of extrathyroidal invasion is made, identification and removal of possibly affected parathyroid glands (as affected lymph nodes or soft tissue) could be attempted [9], as late and incomplete resection of cervical disease affects patient survival in an adverse way.

Parathyroid metastases, unless multiple, have an uncertain clinical impact on patients, as involvement of one parathyroid will not alter serum calcium levels if the other parathyroid glands are functioning appropriately.

This is the first report of parathyroid involvement in cases of FTC. FTC usually metastasizes at distant sites, although large tumors may invade local structures [10]. Among the three reported FTC patients, one exhibited metastatic foci in the parathyroid not related to direct contact, which may represent a hematogenous spread of follicular cancer. Blood supply to the parathyroid glands is mainly derived from the inferior thyroid arteries, and vein and lymph drainage is also made via thyroid gland vessels [11]. The local circulation is not well established, as blood supply in cases of ectopic parathyroid glands depends on their location [12].

## Conclusions

In conclusion, parathyroid involvement in cases with thyroid cancer is rare and possibly underestimated. We report the first three cases of FTC with parathyroid metastases. Locally invasive tumors may invade the parathyroid gland, independent of patient age and tumor size. The clinical significance of such an event has not yet been answered. Multicentre studies are needed to define the true incidence, explore the specific characteristics and evolution of parathyroid metastases, and determine the influence on disease outcome.

## Abbreviations

FTC: follicular thyroid cancer; H&E: hematoxylin and eosin; PTC: papillary thyroid cancer.

## Competing interests

The authors declare that they have no competing interests.

## Authors’ contributions

KPP conceived and coordinated the study. SM, PM, PKI, and LM gathered the data. MB and AC prepared the database and analyzed the data. NF and KM examined the histological material. SM drafted the tables. KPP and AC wrote the manuscript. All authors read and approved the final manuscript.

## Authors’ information

KPP has great experience in the management of patients with endocrine malignancies.
